# Change over time in adolescent smoking, cannabis use and their association: findings from the School Health Research Network in Wales

**DOI:** 10.1093/pubmed/fdaa174

**Published:** 2020-09-29

**Authors:** N Page, B Hallingberg, R Brown, E Lowthian, G Hewitt, S Murphy, G Moore

**Affiliations:** Centre for Development, Evaluation, Complexity and Implementation in Public Health Improvement, School of Social Sciences, Cardiff University, Cardiff, CF10 3BD, UK; Cardiff School of Sport and Health Sciences, Cardiff Metropolitan University, Cardiff, CF5 2YB, UK; Centre for Development, Evaluation, Complexity and Implementation in Public Health Improvement, School of Social Sciences, Cardiff University, Cardiff, CF10 3BD, UK; Centre for Development, Evaluation, Complexity and Implementation in Public Health Improvement, School of Social Sciences, Cardiff University, Cardiff, CF10 3BD, UK; Centre for Development, Evaluation, Complexity and Implementation in Public Health Improvement, School of Social Sciences, Cardiff University, Cardiff, CF10 3BD, UK; Centre for Development, Evaluation, Complexity and Implementation in Public Health Improvement, School of Social Sciences, Cardiff University, Cardiff, CF10 3BD, UK; Centre for Development, Evaluation, Complexity and Implementation in Public Health Improvement, School of Social Sciences, Cardiff University, Cardiff, CF10 3BD, UK; SPECTRUM Consortium, UK

**Keywords:** cannabis, public health, smoking, young people

## Abstract

**Background:**

While tobacco smoking has declined among UK youth in recent decades, cannabis use has begun to show some growth. Given their interrelationship, growth in cannabis use may act as a barrier to continued reduction in youth smoking. This paper assesses recent tobacco and cannabis use trends in Wales, and their association, to explore whether change in cannabis use might have impacted youth tobacco smoking prevalence.

**Methods:**

Repeat cross-sectional data on tobacco and cannabis use were obtained from biennial Welsh Student Health and Wellbeing surveys between 2013 and 2019. Data were pooled and analysed using logistic regression with adjustment for school-level clustering.

**Results:**

No change in regular youth tobacco smoking was observed between 2013 and 2019. In contrast, current cannabis use increased during this time, and cannabis users had significantly greater odds of regular tobacco smoking. After adjusting for change in cannabis use, a significant decline in youth tobacco smoking was observed (OR 0.95; 95% confidence intervals: 0.92, 0.97).

**Conclusion:**

Recent growth in cannabis use among young people in Wales may have offset prospective declines in regular tobacco smoking. Further reductions in youth smoking may require more integrated policy approaches to address the co-use of tobacco and cannabis among adolescents.

## Introduction

Tobacco use remains a leading driver of morbidity, mortality and health inequalities.^1^ Globally, it is the second largest determinant of disability-adjusted life years, and the leading determinant among many western European countries.^2^ Earlier age of smoking initiation is linked with greater severity of harm from tobacco use,^3^ hence tobacco control strategies continue to target prevention efforts at children and adolescents. UK approaches have included raising the legal age of purchase to 18, restricting point-of-sale advertising, and introducing plain packaging for tobacco products.^4^ In combination, these policies are part of two decades of work to de-normalize smoking, alongside major policy moves through banning marketing and smoking in enclosed public spaces. The advancement of restriction and de-normalization within tobacco control policy^5^ has achieved substantial population-level reductions in adolescent smoking uptake. A recent pan-European study reported rates of daily use among 15–16 year olds halved in western Europe between 1999 and 2015.^6^ UK survey data support this trend, with substantial declines in youth smoking uptake having occurred over the last two decades.^7^

However, recent figures from both England and Wales suggest declines in regular youth smoking may be slowing; prevalence rates among 11–15 year olds have remained relatively consistent since 2013, at around 2–3% in England and 4% (among 11–16 year olds) in Wales.^8^^,^^9^ Prevalence data from Scotland indicate a similar trend with regular smoking among 13 and 15 year olds showing no change since 2015.^10^ Tobacco de-normalization has reduced smoking uptake and stigmatized smoking,^11^ increasingly positioning it as a ‘deviant’ behaviour. Thus, the minority of young people who continue to take up smoking do so despite its growing de-normalization, or perhaps in active rejection of these norms. Previous research, for example, has linked rejection of certain school-based norms (e.g. authoritarian, attainment-driven cultures) with the adoption of behaviours framed as deviant as alternative markers of status, bonding and identity.^12^ Hence, this plateauing in part may indicate that declines are reaching the limits of what can be achieved through de-normalization approaches, and that other strategies may be needed to reach those who continue to take up smoking.

Declines in tobacco use observed in the past two decades in the UK have until recently been mirrored by declines in use of other substances such as alcohol and cannabis.^7^ However, there is some indication that cannabis use is now growing: the percentage of adolescents in England reporting past month use of cannabis increased from 3.9 to 4.4% between 2014 and 2018, while use the previous 12 months increased from 6.7 to 8.8%.^8^ As in many European countries, use of cannabis is illegal in the UK, although cannabis-based products can now be prescribed for medicinal purposes,^13^ while international approaches to cannabis regulation have moved in the direction of greater liberalization.^14^ Among UK adolescents, self-report data suggest cannabis is the most commonly used illicit substance.^8^^,^^9^ Unsurprisingly, both prevalence and frequency of cannabis use have been inversely associated with perceived risk among adolescents^15^; and there is some evidence that increased liberalization of cannabis regulation might negatively impact cannabis harm perceptions among young people.^16^ However, to date there is little evidence that greater liberalization increases youth use of cannabis.^17^

Evidence points towards a strong association between tobacco and cannabis, with use of both substances frequently shown to co-occur.^18–20^ Co-use commonly refers either to singular use of both tobacco and cannabis or co-administration, e.g. via rolled cigarettes containing both tobacco and cannabis (‘spliffs’), with some authors classifying co-use as past month use of both substances.^19^^,^^20^ Potential mechanisms underlying the association between tobacco and cannabis have been suggested to include shared genetic or environmental factors (e.g. exposure/availability), or direct causation via gateway (tobacco use leads to cannabis use) or reverse gateway (cannabis use leads to tobacco use) effects.^21–23^ The sharing of a common route of administration (i.e. inhalation) may also be impactful with smoked, but not smokeless, tobacco having been associated with increased risk for cannabis use, adjusting for nicotine dependence and other covariates.^24^ In Europe, co-administration of tobacco and cannabis is the most common means of cannabis consumption, with cannabis users favouring this approach over other methods, such as edibles or non-combustibles (e.g. vaporizers).^25^ Supplementation with tobacco reduces the amount of cannabis required, making it less costly, while co-administration has also been shown to increase the amount of delta-9-tetrahydrocannabinol (THC; the main psychoactive component within cannabis) inhaled per gram of cannabis.^26^

Given this proclivity for co-administration among Europeans, it is highly likely that change in prevalence of one substance will greatly impact rates of use of the other. Hence growth in cannabis use, given its substantial overlap with tobacco use, may be acting as a significant barrier to further reducing youth smoking. The continued positioning of a once normalized behaviour (smoking) as deviant, coupled with the increasing normalization of a previously deviant behaviour (cannabis use),^27^ may equally be leading a convergence of tobacco and cannabis use behaviours among the same young people. Investigation of current tobacco and cannabis use trends, and their association, are therefore warranted within a UK context to assess the extent to which stalling declines in youth tobacco smoking uptake are linked with increasing youth use of cannabis.

This paper draws upon nationally representative survey data from secondary school-aged children in Wales, UK, to examine associations between tobacco and cannabis use to explore whether change in cannabis use might have impacted youth tobacco smoking trends. The paper first examines temporal changes in prevalence of tobacco smoking and cannabis use between 2013 and 2019, before examining the overlap between tobacco smoking and cannabis use at each time point. The paper then assesses whether adjusting for change in cannabis use over the study period substantially altered the time trend for tobacco smoking. Implications for policy borne from these results are then discussed.

## Methods

### Survey instrument

Repeat cross-sectional data were obtained from the 2013 Welsh Health Behaviour in School-aged Children (HBSC) survey and 2015, 2017 and 2019 School Health Research Network (SHRN) Student Health and Wellbeing (SHW) surveys. The SHW survey is modelled on the HBSC Wales survey with the latter (undertaken every 4 years) nested within SHRN’s biennial SHW survey since 2017, allowing samples to be pooled for statistical analyses. Administered within Welsh secondary schools, the questionnaire is completed by a core sample of 11–16 year olds following the obtainment of informed consent from schools, parents and students. The 2013 survey obtained data from 82 schools; a school response rate of 46%. By 2019, SHRN membership included all maintained secondary schools and middle schools in Wales, of which students from 94% of schools (*n* = 198) completed the SHW survey. The 2013 survey was paper-based, while the 2015, 2017 and 2019 surveys were delivered electronically. More detailed descriptions of survey methodologies can be found elsewhere.^9^^,^^28^

## Measures

### Socio-demographics

Students reported their gender (boy, girl or neither word describes me: the latter an additional response option introduced in 2019) and school year (UK school years 7–11). A binary measure of socioeconomic status (SES) was derived using the Family Affluence Scale (FAS), which comprises a number of indicators of material affluence including bedroom occupancy, car and computer ownership and family holidays.^29^ Items were summed to form a scale score and high/low SES was defined based on whether scores were above/below the FAS median score for the survey year.

### Tobacco smoking

A binary measure of regular smoking was derived from the question; ‘How often do you smoke tobacco at present?’. Responses ‘every day’ or ‘at least once a week, but not every day’ were classified as smoking regularly and compared to responses ‘less than once a week’ or ‘I do not smoke’.

### Cannabis use

No weekly measure of cannabis use was available, so a binary measure indicating current cannabis use was derived from the question; ‘Have you ever taken cannabis in the last 30 days?’. Any response indicating past month use of cannabis (ranging from ‘1 to 2 days’ to ‘30 days or more’) was classified as current use.

### Analysis

First, change in prevalence of both tobacco and cannabis use measures over time was explored descriptively. Binary logistic regression was then used to model trends in smoking and cannabis use between 2013 and 2019, controlling for gender, school year and SES, with adjustment for school-level clustering. Additional subgroup analyses by gender and SES were also explored. Initially, cannabis use as a predictor of tobacco smoking was modelled separately for each time point (with demographic controls) to explore any change in association between measures over the four cross-sections, before adjusting for cannabis use in the 2013–2019 model and associated subgroup models. Inclusion of a time^*^cannabis use interaction term was also modelled to explore changes in the strength of the relationship between cannabis use and tobacco smoking over time. For the purpose of this analysis, students who stated neither gender best described them were excluded from the pooled sample given that this categorization was only introduced in 2019 and thus temporal change in tobacco and cannabis use among this subgroup could not be examined; gender non-response was however included, but not modelled within subgroup analyses. Survey weights were available for the 2013 HBSC data to correct for biases associated with survey design and non-response. However, following a comparison of 2013 weighted and unweighted sample demographics, the decision was taken not to apply these weights within our primary statistical analyses. Age breakdown and SES in the 2013 sample were shown to be more comparable to the much larger 2017 and 2019 samples (which closely mirrored the equivalent population of Wales with responses collected from over 90% of eligible secondary schools) prior to weighting. Weighted regression models were however estimated (where weighting values for 2015, 2017 and 2019 surveys were set to 1) as a sensitivity analysis and any significant variation in results reported. In this study, all statistical analysis was undertaken using Stata v.15.1.

### Research ethics

Biennial HBSC and SHRN surveys were each approved by the Cardiff University School of Social Sciences Ethics Committee.

## Results

The total pooled sample consisted of 263 333 responses; 9055 from 2013, 32 391 from 2015, 103 971 from 2017 and 117 916 from 2019. Sample breakdown by survey year is provided in Table 1. For the overall pooled sample, breakdown by student socio-demographics and health behaviours revealed an even distribution of responses from adolescent boys (48.9%) and girls (49.9%), with only a small proportion of gender non-response (1.3%). Similarly well distributed were responses by school year (year 7: 22.1%; 8: 21.6%; 9: 20.7%; 10: 18.9% and 11: 16.7%) and SES (low SES: 50.2%). Of those students who provided responses to questions on tobacco and cannabis use, 3.7% reported regular (weekly) tobacco smoking and 4.1% current (past month) cannabis use. Co-use of both tobacco and cannabis was high, with 51.5% of current cannabis users reporting regular tobacco smoking. Only 1.3% of students who were not current cannabis users were regular smokers.

**Table 1:** Sample characteristics, 11–16 year olds participating in Welsh HBSC/SHRN SHW surveys between 2013 and 2019

	*Survey year*			
	*2013^a^*	*2015*	*2017*	*2019*
Gender, *n* (%)				
Boy	4565 (50.4)	15 554 (48.0)	50 452 (48.5)	58 115 (49.3)
Girl	4457 (49.2)	16 837 (52.0)	51 458 (49.5)	58 610 (49.7)
Did not state	33 (0.4)	0 (0.0)	2061 (2.0)	1191 (1.0)
School year				
Year 7	1893 (20.9)	7208 (22.3)	22 634 (21.8)	26 550 (22.5)
Year 8	1879 (20.8)	7008 (21.6)	22 421 (21.6)	25 541 (21.7)
Year 9	1897 (21.0)	6449 (19.9)	22 208 (21.4)	24 054 (20.4)
Year 10	1778 (19.6)	6358 (19.6)	19 704 (19.0)	21 866 (18.5)
Year 11	1608 (17.8)	5368 (16.6)	17 004 (16.4)	19 905 (16.9)
FAS				
High	4040 (46.0)	13 531 (47.3)	51 770 (49.8)	56 200 (50.7)
Low	4739 (54.0)	15 081 (52.7)	52 201 (50.2)	54 610 (49.3)
Smoking (weekly)				
Yes	299 (3.3)	1126 (3.7)	3835 (3.8)	4061 (3.7)
No	8685 (96.7)	29 337 (96.3)	96 170 (96.2)	106 883 (96.3)
Cannabis use (past month)				
Yes	249 (2.7)	756 (2.8)	4305 (4.4)	4772 (4.3)
No	8806 (97.3)	26 093 (97.2)	94 156 (95.6)	105 254 (95.7)

^a^HBSC Wales survey.

Over the time series, prevalence of regular tobacco smoking was 3.3% (95% confidence intervals [CI]: 3.0–3.7%) in 2013 and 3.9% (95% CI: 3.8–4.0%) in 2019, with no evidence of a statistically significant change over this period (Model 1). Comparatively, prevalence of current cannabis use increased from 2.7% (95% CI: 2.4–3.1%) to 4.6% (95% CI: 4.4–4.8%) over this time, with significant growth in overall use (Model 2) and across all subgroups (Models 2a–d) equivalent to a biennial increase in odds of regular use of 8% (Table 2). Cannabis use was associated with significantly increased odds of smoking at each time point: compared to non-users, cannabis users consistently had greater odds of being regular smokers, rising from 39.5 (95% CI: 27.6–56.7) times greater odds in 2013 to 55.9 (95% CI: 50.1–62.3) times greater odds in 2019.

**Table 2:** Odds ratios (and 95% CI) for change over time in both regular smoking and current cannabis use among students in Wales between 2013 and 2019, overall and by gender and SES

		*Tobacco smoking*		*Cannabis use*	
		*OR*	*P*	*OR*	*P*
Total (*n* = 241 626; *n* = 236 364)		Model 1		Model 2	
	Time (year)	1.01 [0.99, 1.03]	0.488	1.08 [1.05, 1.10]	<0.001
	Gender				
	Female	0.97 [0.91, 1.03]	0.316	0.76 [0.71, 0.80]	<0.001
	Did not state	5.39 [4.69, 6.20]	<0.001	3.90 [3.38, 4.51]	<0.001
	School year	1.85 [1.81, 1.89]	<0.001	2.07 [2.02, 2.13]	<0.001
	Low SES	1.46 [1.37, 1.54]	<0.001	1.08 [1.05, 1.10]	<0.001
Boys (*n* = 117 314; *n* = 114 577)		Model 1(a)		Model 2(a)	
	Time	1.02 [0.99, 1.05]	0.218	1.08 [1.06, 1.11]	<0.001
	School year	1.78 [1.73, 1.82]	<0.001	2.03 [1.97, 2.10]	<0.001
	Low SES	1.40 [1.30, 1.51]	<0.001	1.09 [1.02, 1.16]	0.011
Girls (*n* = 121 951; *n* = 119 459)		Model 1(b)		Model 2(b)	
	Time	1.00 [0.97, 1.04]	0.796	1.08 [1.05, 1.11]	<0.001
	School year	2.00 [1.94, 2.06]	<0.001	2.20 [2.13, 2.28]	<0.001
	Low SES	1.51 [1.40, 1.63]	<0.001	1.19 [1.10, 1.28]	<0.001
High SES (*n* = 120 494; *n* = 117 896)		Model 1(c)		Model 2(c)	
	Time	1.02 [0.99, 1.05]	0.221	1.08 [1.05, 1.11]	<0.001
	Gender				
	Female	0.93 [0.85, 1.01]	0.075	0.72 [0.67, 0.78]	<0.001
	Did not state	5.21 [4.29, 6.32]	<0.001	3.87 [3.16, 4.74]	<0.001
	School year	1.83 [1.77, 1.89]	<0.001	2.16 [2.09, 2.23]	<0.001
Low SES (*n* = 121 132; *n* = 118 468)		Model 1(d)		Model 2(d)		
	Time	1.00 [0.98, 1.03]	0.958	1.08 [1.05, 1.11]	<0.001	
	Gender					
	Female	1.00 [0.93, 1.07]	0.993	0.79 [0.73, 0.85]	<0.001	
	Did not state	5.53 [4.54, 6.74]	<0.001	3.94 [3.23, 4.79]	<0.001	
	School year	1.86 [1.81, 1.91]	<0.001	2.00 [1.94, 2.07]	<0.001	

OR: odds ratio.

After controlling for cannabis use (alongside gender, school year and SES) in our regression model (Model 3), a significant decline in regular tobacco smoking emerged, equal to a 5% biennial reduction in odds (Table 3). This is consistent with a hypothesis that had there been no change in cannabis use, smoking rates would be expected to decline. In subgroup analyses (Models 3a–d), estimates indicated a similar size decrease in relative odds for both boys and girls and students of high or low SES following adjustment for cannabis use. Inclusion of a time^*^cannabis use interaction term (Model 4) to model changes in the strength of the relationship between cannabis and smoking over time was not statistically significant, although neared significance in the all-girl model (Model 4b). Here, additional *post-hoc* analysis revealed that, among girls, the percentage of current cannabis users who were also regular smokers declined by 12.9% points over the time series (from 64.7% in 2013 to 51.8% in 2019), suggesting a growth in uptake of cannabis use among non-smoking girls.

**Table 3:** Odds ratios (and 95% CI) for regular smoking among students in Wales between 2013 and 2019, overall and by gender and SES

		*OR*	*P*	*OR*	*P*
Total (*n* = 231 033)		Model 3		Model 4	
	Time (year)	0.95 [0.92, 0.97]	<0.001	0.95 [0.92, 0.98]	0.001
	Gender				
	Female	1.19 [1.11, 1.28]	<0.001	1.19 [1.11, 1.28]	<0.001
	Did not state	4.06 [3.39, 4.87]	<0.001	4.06 [3.38, 4.86]	<0.001
	School year	1.42 [1.38, 1.46]	<0.001	1.42 [1.38, 1.46]	<0.001
	Low SES	1.55 [1.45, 1.66]	<0.001	1.55 [1.45, 1.66]	<0.001
	Cannabis use	57.45 [52.82, 62.49]	<0.001	57.49 [52.84, 62.54]	<0.001
	Time^*^cannabis	—	—	0.99 [0.95, 1.03]	0.570
Boys (*n* = 111 787)		Model 3(a)		Model 4(a)	
	Time	0.95 [0.92, 0.98]	0.003	0.94 [0.91, 0.98]	0.002
	School year	1.32 [1.28, 1.37]	<0.001	1.32 [1.28, 1.37]	<0.001
	Low SES	1.50 [1.38, 1.63]	<0.001	1.50 [1.38, 1.63]	<0.001
	Cannabis use	55.78 [50.35, 61.79]	<0.001	55.69 [50.25, 61.72]	<0.001
	Time^*^cannabis	—	—	1.03 [0.97, 1.09]	0.319
Girls (*n* = 117 087)		Model 3(b)		Model 4(b)	
	Time	0.94 [0.91, 0.98]	0.001	0.96 [0.92, 1.00]	0.047
	School year	1.56 [1.50, 1.62]	<0.001	1.56 [1.50, 1.62]	<0.001
	Low SES	1.62 [1.48, 1.77]	<0.001	1.62 [1.48, 1.77]	<0.001
	Cannabis use	59.02 [52.79, 65.98]	<0.001	59.29 [53.02, 66.30]	<0.001
	Time^*^cannabis	—	—	0.94 [0.89, 1.00]	0.055
High SES (*n* = 115 354)		Model 3(c)		Model 4(c)	
	Time	0.96 [0.93, 0.99]	0.022	0.96 [0.92, 1.01]	0.091
	Gender				
	Female	1.15 [1.04, 1.27]	0.005	1.15 [1.04, 1.27]	0.005
	Did not state	4.13 [2.99, 5.71]	<0.001	4.13 [2.99, 5.70]	<0.001
	School year	1.34 [1.29, 1.40]	<0.001	1.34 [1.29, 1.40]	<0.001
	Cannabis use	57.35 [50.92, 64.60]	<0.001	57.40 [50.96, 64.67]	<0.001
	Time^*^cannabis	—	—	0.99 [0.93, 1.05]	0.744
Low SES (*n* = 115 679)		Model 3(d)		Model 4(d)	
	Time	0.94 [0.91, 0.97]	<0.001	0.94 [0.91, 0.98]	0.001
	Gender				
	Female	1.22 [1.12, 1.32]	<0.001	1.22 [1.12, 1.32]	<0.001
	Did not state	4.04 [3.24, 5.03]	<0.001	4.03 [3.24, 5.02]	<0.001
	School year	1.47 [1.42, 1.52]	<0.001	1.47 [1.42, 1.52]	<0.001
	Cannabis use	58.17 [52.55, 64.38]	<0.001	58.20 [52.57, 64.43]	<0.001
	Time^*^cannabis	—	—	0.98 [0.93, 1.04]	0.550

OR: odds ratio.

Overall, all findings presented were robust to modelling assumptions with no substantial variation in reported associations between unweighted and 2013 weighted analyses.

## Discussion

### Main finding of this study

In our analysis of Welsh survey data, we observed no change in overall rates of regular tobacco smoking among 11–16 year olds between 2013 and 2019. This finding was consistent for males and females and those of higher or lower affluence. Comparatively, the proportion of adolescents reporting current use of cannabis increased over the study period, with growth equating to an 8% biennial increase in odds of current use. Consistent with existing evidence, a strong association between tobacco and cannabis use was demonstrated and adjusting for cannabis use in our regression model led to a significant decline in tobacco smoking prevalence over time, reversing the previous finding of no change since 2013. Taken together, these findings are consistent with a hypothesis that, for Wales, recent plateauing in youth tobacco smoking rates is associated with simultaneous growth in cannabis use. These findings intimate that had rates of cannabis use remained constant over time, prevalence rates for tobacco smoking might have continued to fall among adolescents in Wales.

### What is already known on this topic

Population-level declines in tobacco smoking have occurred in many countries following increased tobacco control over the last two decades,^6^ with the ban on smoking in enclosed public spaces recently voted the most important public health achievement of the 21st century by the Royal Society for Public Health UK.^30^ However, the de-normalization of smoking brought about by greater tobacco control contrasts with seemingly more permissive norms towards cannabis in international policy; demonstrated by the legalization of medicalized cannabis,^13^ changing public attitudes regarding its harmfulness,^31^ more liberal policing of small quantities,^32^ and evidence of a recent growth in recreational use among young people.^8^ Given well-established links between tobacco and cannabis, and the common trend towards co-administration among European populations, it is likely that change in prevalence of one substance will impact on rates of use of the other.

### What this study adds

This study highlights a need to consider the strong interrelationship between tobacco smoking and cannabis use in youth smoking cessation strategies. In Wales, existing tobacco control policy has targeted a 5% weekly smoking rate among 15–16 year olds by 2020, down from 9% in 2013–2014.^33^ It seems unlikely that this target will be achieved if cannabis use continues to grow, and coordinated efforts to address tobacco and cannabis use uptake and their co-occurrence among young people should therefore be prioritized to help in achieving this target. Further, co-use of tobacco and cannabis has been shown to lead to poorer smoking cessation outcomes,^34^ and while nicotine replacement and behavioural support are well-evidenced cessation aids available to regular youth smokers,^35^ youth cessation programmes do not currently address co-use of cannabis; although the feasibility of implementing such an integrated programme has been explored.^36^ In the UK, longitudinal evidence shows occasional (early-onset, late-onset) and regular cannabis users have significantly greater odds of nicotine dependence at age 21, compared to non-cannabis users.^18^ Thus, if cannabis use is a key motivation for tobacco smoking among youth, as has been proposed,^37^ failing to consider this relationship may constitute an unmet need within current cessation services. Policy-makers are therefore advised not to consider smoking in isolation, but as part of a broader pattern of adolescent substance use and risk taking.^38^ Continued reductions in youth smoking may require more integrated approaches to prevention and cessation with existing drug policy.

### Limitations of this study

There are a number of limitations to this study that must be noted. First, while our tobacco measure specified smoking, the mechanism of cannabis delivery (e.g. smoking, edibles or vaping) was not specified, which could have impacted student response. Second, the SHW survey asks students to report their tobacco and cannabis use separately and therefore we cannot comment on rates of co-administration among young people in Wales, and whether this practice could be driving the plateau in smoking rates. Third, tobacco and cannabis measures reflect use over different periods of time (‘weekly’ versus ‘past month’). However, while use of cannabis in the last 30 days was used as a best alternative due to no weekly measure being available, this measure would still be largely expected to capture current use rather than experimentation—corresponding with our measure of tobacco smoking. Fourth, self-report data are limited by social desirability biases, which may themselves change over time as behaviours become more or less normalized. Further, use of repeat cross-sectional data means causal inference cannot be made regarding the nature of the association between tobacco and cannabis use within our sample; longitudinal data are required to better understand the complex interplay over time. As such, this paper presents preliminary analysis which will inform longitudinal work into the interconnection between tobacco smoking and cannabis use as longitudinal capability is built into the full SHW survey from 2019. Finally, it is possible that change in mode of delivery from a paper to electronic survey could have resulted in some bias—although a similar adolescent health survey found no evidence of such a change affecting survey representativeness or responses to substance use questions.^39^

## Conclusion

Given the interrelationship between tobacco and cannabis use,^21^^,^^40^ and the appetite for co-administration use among European populations,^25^ this study explored recent trends in use of both substances, and their association, among a large sample of adolescents in Wales, to assess the extent to which stalling declines in youth smoking uptake in the UK may be linked to changes in prevalence of youth use of cannabis. Overall, despite evidence of no change in regular youth tobacco smoking prevalence since 2013, this was reversed after adjustment for growth in cannabis use over the study period, suggesting recent growth in cannabis use may have offset prospective declines in youth tobacco smoking in Wales.

## Conflict of interest

None declared.

## Funding

The Centre for Development, Evaluation, Complexity and Implementation in Public Health Improvement (DECIPHer) is funded by Welsh Government through Health and Care Research Wales. This work was supported also by the Public Health Division, Welsh Government with the support of The Centre for the Development and Evaluation of Complex Interventions for Public Health Improvement (DECIPHer), a UKCRC Public Health Research Centre of Excellence. Joint funding (MR/KO232331/1) from the British Heart Foundation, Cancer Research UK, Economic and Social Research Council, Medical Research Council, the Welsh Government and the Wellcome Trust, under the auspices of the UK Clinical Research Collaboration, is gratefully acknowledged. The School Health Research Network is a partnership between DECIPHer at Cardiff University, Welsh Government, Public Health Wales and Cancer Research UK, funded by Health and Care Research Wales.

## Notes

**N. Page,** Research Associate

**B. Hallingberg,** Lecturer in Health and Wellbeing

**R. Brown,** Research Associate

**E. Lowthian,** PhD Student

**G. Hewitt,** Research Associate

**S. Murphy,** Professor in Social Interventions and Health

**G. Moore,** Professor
